# Safety and Performance of Titanium Suture Anchors Used in Knee Ligament Repair Procedures

**DOI:** 10.3390/medicina57030287

**Published:** 2021-03-19

**Authors:** Antonio Maestro, Iván Pipa, Nicolás Rodríguez, Carmen Toyos, Marcelino Torrontegui-Duarte, Cesar Castaño

**Affiliations:** 1 Hospital Begoña, Avda, Pablo Iglesias 92, 33204 Gijón, Spain; doctorantoniomaestro@gmail.com (A.M.); pipa85@msn.com (I.P.); rodriguezgcia@gmail.com (N.R.); carmentoyos@hotmail.com (C.T.); 2 Real Sporting Gijon SAD, Camino Mareo-Granda, 645, 33390 Gijon, Spain; cesarcastanofernandez@gmail.com; 3 Departament Nursing and Podiatry, Universidad de Malaga, 29016 Malaga, Spain

**Keywords:** titanium, suture anchor, knee, ligament, anterolateral

## Abstract

Injuries to the knee ligaments can be particularly disabling in young patients, given the risk of long-term disability if adequate fixation is not achieved during initial repair. The TWINFIX™ titanium (Ti) suture anchor with ULTRABRAID™ Suture (Smith and Nephew, London, UK) was designed to secure tendon and ligament reconstructions with increased boney ingrowth at the anchor site with minimal invasive technique. This retrospective analysis looked at 33 patients (41 implants) operated with this device between 2015 and 2019 at a single institution. The average age of patients was 33.18 years (standard deviation [SD], 15.26), with an average body mass index of 24.88 (SD, 3.49). The indications were lateral extra-articular tenodesis during anterior cruciate ligament reconstruction, medial patellofemoral ligament reconstruction, quadriceps or patellar tendon repair and medial collateral ligament repair. After an average follow up of 24.3 + 6.53 months, there was no reports of clinical failure or radiographic evidence of implant failure or loosening. One patient experienced a complication unrelated to the study device, requiring manipulation under anesthesia with resolution of symptoms. This case series supports the safety and performance of this implants for the knee procedures in which its use is indicated. Additional follow-up will be required to determine whether these effects are sustained at medium- and long-term durations.

## 1. Introduction

Although suture anchors are an established therapeutic option within orthopaedic [1] and sports medicine [2] procedures, there continues to be a need for newer and more advanced anchor systems to secure tendon [3] and ligament reconstructions with increased boney ingrowth at the anchor site [4]. This is particularly important in highly active, relatively younger patients, or those who want or need an early return to job or activities [5], who may benefit from less-invasive reconstruction surgeries (almost percutaneous) that offer greater biological and mechanical security.

There are numerous indications for suture anchors in knee ligament repair procedures [6], including quadriceps tendon repair [7,8,9,10], extracapsular medial patellofemoral ligament (MPFL) reconstruction [11], or fracture fixation [12,13]. Especially when the anchors are used, the sutures must carry out the requirement to maintain their hardness in the initial phase of the process (early post-operatory), in order to initially act through mechanical resistance and allow the biological healing of the tissues.

Emerging trends for anterior cruciate ligament (ACL) reconstruction involves adding a lateral extra-articular tenodesis (LET) or anterolateral ligament reconstruction [14] since this technique pursues a restrain of the medial tibial torsion adding some soft tissue, as well as patellar dislocation with minimal invasive techniques [14,15,16] instead of open or more aggressive conventional surgical techniques.

The TWINFIX™ titanium (Ti) suturanchor with ULTRABRAID™ Suture (Smith and Nephew, London, UK) was designed to offer additional strength and stability over traditional suture systems.

While there have been studies evaluating the advantages and disadvantages of various fixation methods for Knee Ligament Reconstruction [17,18], no clinical studies have evaluated the efficacy of the use of these kind of implants in knee surgery on the techniques above related in adults and no evidence was publish until nowadays.

We hypothesized that the use of these anchors or fixation’s methods are the ideal treatment method or at least one more therapeutic option for providing stable fixation and satisfactory clinical outcomes after a 6-months minimal follow-up. The purpose of the study was (1) to describe arthroscopic anchor suture-bridge fixation as a novel technique for treating tibial intercondylar eminence fractures in adults; and (2) to analyze the minimal follow-up results of the radiographic and clinical outcomes.

## 2. Materials and Methods

### 2.1. Subjects, Demographic Data and Surgery

From July 2015 (after introduction of the device) to December 2019, 33 consecutive patients underwent knee ligament repair procedures with the study device at a single hospital center. Patients who met clinical criteria for operative intervention had the following injuries and indications: ACL reconstruction with anterolateral reconstruction, extra-capsular MPFL reconstruction, quadriceps or patellar tendon repair, and medial collateral ligament (MCL) repair. All included patients were evaluated by a sports medicine fellowship trained orthopaedic surgeon and operated on by the same hospital team.

The average age of patients was 33.18 years (standard deviation, 15.26), with 10 under the age of 21. Patients had an average body mass index of 24.88 (standard deviation, 3.49) and were followed for an average of 24.3 months (standard deviation, 6.53) postoperatively.

The study protocol was in accordance with standard ethical and human research principles. Written informed consent for participation and publication was given by a parent of each participant, including the publication of photographs. The study was approved by the Research Ethics Committee of the Principado of Asturias (2020/257, approved date: 26 February 2020), Spain.

All patients received same devices of Titanium, preloaded with the ultrabraid suture for soft tissue attachment in the bone and had signs of knee joint instability (between femur and tibia or between femur and patella) or functional insufficiency on quadriceps or patellar tendons prior to the procedure. Once the implant was placed inside the bone, there are two possibilities or direct fixations by means of a loop with the knot on the ligament itself or by means of a suture to the ligament according to the Krackow technique [19] in order to betray the ligament and anchor it in contact with the bone. Follow-up included ensuring functional stability, functional recovery exploring flexion and extension degrees (and compared with non-injured knee) and radiological studies (Antero-posterior (AP), lateral and axial views) were made postoperatively, and at 18 months.

### 2.2. Outcomes Measures

The primary outcome was implant failure, which was defined as any implant with one of the following three conditions: clinical instability used the Knee Injury and Osteoarthritis Outcome Score (KOOS). The KOOS comprises 42 items in 5 separately scored subscales assessing pain and function of the knee in patient with injury or osteoarthritis [20].

The subscales are divided in Pain (nine items); Symptoms (seven items); Function in daily living (ADL) (17 items); Sport and Recreation Function (Sport/Rec) (five items); Quality of Life (QoL) four items. Each item is rated on a 0- to 4-Likert scale, and each of the five subscales is calculated as the sum of the items included. Scores are then transformed to a 0–100 scale. The measure generates five separate scores where the higher the score, the best the health state.

The presence or absence of local inflammatory signs, stiffness or pain, as well as general patient variables such as age, sex and body mass index, were recorded as secondary variables. The participant was asked to return after 6 months and then score the KOOS he or she had perceived during the previous 3 days.

### 2.3. Statistical Analysis

Data were analyzed with the IBM SPSS 24^®^ (SPSS Science, Chicago, IL, USA). The Shapiro–Wilk test applied to the data showed the distribution to be normal in the pre and postoperative evaluation. It was therefore decided to use Student’s *t*-test for paired samples to compare Pre-Post and the effect size by Cohen’s d. Differences were considered to be statistically significant if *p* < 0.05.

## 3. Results

**Figure 1:**
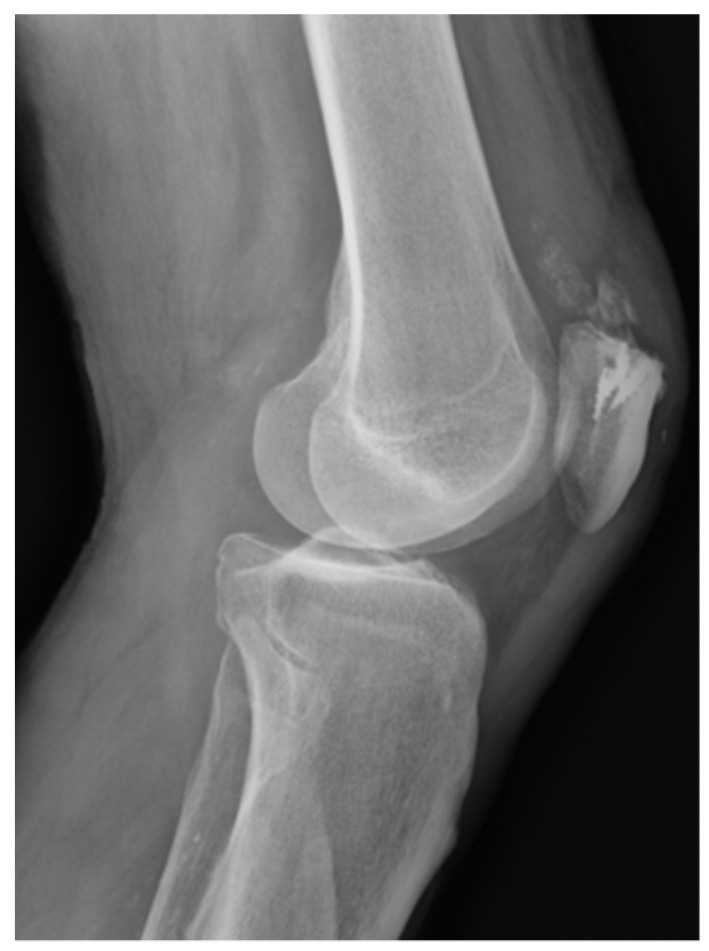
Quadriceps reconstruction after its rupture by mean of 4 TWINFIX.

Thirty-three patients (41 implants) underwent operative intervention with procedures that required the implantation of a suture anchor. Of the 33 patients, 27 had one implant (27 implants), five had two implants (10 implants), and one had four implants (Figure 1).

**Figure 2:**
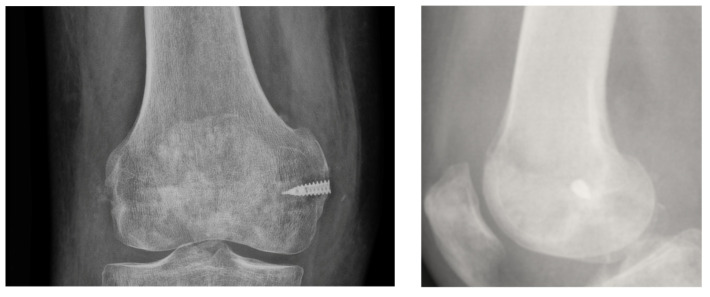
TWINFIX on the epicondyle footprint for medial collateral ligament, at the time of Anterior Cruciate Ligament ACL reconstruction.

Indications for surgery included 24 patients with LET during Anterior Cruciate Ligament (ACL) reconstruction (in anterolateral margin of the proximal tibia at half of distance between head of fibula and Gerdy´s tubercle), two patients with extra-capsular MPFL reconstruction (on the medial margin of the patella´s border), three patients with quadriceps or patellar tendon repair (inside of the respective bone), and four with MCL repair (on the medial aspect of the proximal tibia, or close to the epicondyle targeting the anatomic footprint of MCL) (Figure 2).

All 33 patients had postoperative radiographs, which demonstrated visible implant tracks in the knee. There was no evidence on initial or repeat imaging, or upon clinical examination, of implant failure, dislocation, or loosening in any patient.

The mean preoperative and of post- operative KOOS score are shown in Table 1 (*p* < 0.001).

There were three reported non-serious adverse events after ACL reconstruction: two cases of minor dysesthesia in the knee and one case of femoropatellar pain. One patient had to return to the hospital for two days because of fever, stiffness, and swelling one week after surgery was performed. No infection was diagnosed, and the patient’s symptoms resolved with medication only.

Only one patient had complications that required return to the operating room. The patient had a post-operative local infection, swelling, and stiffness, which required manipulation under anesthesia. He subsequently developed lateral inflammation above the anterolateral ridge and required antibiotics, cleaning of the wound, and small drainage by means of one minimum incision. This resulted in resolution of symptoms.

None of the complications mentioned above were considered to be related to the study device.

## 4. Discussion

The most important finding of this study was to know the possibility for using these types of metallic implant in knee surgery for ligaments or tendons reconstructions, and the no presence of adverse effects. These Suture Anchors are designed to provide secure reattachment of soft tissue to bone and facilitate bone ingrowth. Attachment of soft tissue is performed with the surgeon’s preferred technique. These devices can be used with instruments such as drills, threaded dilators, and awls.

**Figure 3:**
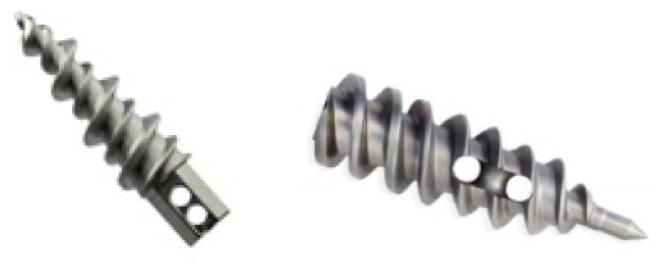
The TWINFIX designed devices. (See footprint selection on the text).

The body of these anchor products is fabricated from ASTM F-136 titanium alloy. Both anchors come preloaded with a non-absorbable suture. All sutures are made of ultra-high molecular weight (UHMW) polyethylene fiber. In terms of mechanical design, the primary difference between the two anchors relate to eyelet placement. The Ultra Ti suture anchor contains a distal suture eyelet that accommodates internal suture routing, enabling anchor cortical fixation. The Ti suture anchor contains a proximal suture eyelet that does not afford anchor thread engagement with the cortical layer (Figure 3).

**Figure 4:**
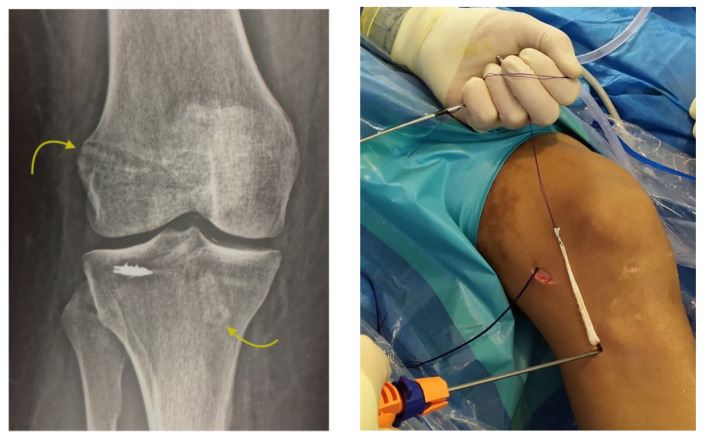
Anterolateral fixation of the plasty at the time of lateral extra-articular (LET) (see footprint selection on the text). Arrows show femur and tibia tunnels with two interence screws fixation across out-in technique. Minimally invasive surgery for introducing the TWINFIX into the bone is showed.

Approximately 97% of extra-capsular repair cases with LET combined with ACL reconstruction relied on implants placed on the lateral tibial surface at a location point situated halfway between the fibular head and the Gerdy´s tubercle by means of a minimally invasive incision (Figure 4).

There was no complication related to the use of this implant in the knee joint procedures. Occurrence of patient complications such as joint rigidity (slow return of range of motion) was entirely related to healing of the surgical joint incision and not related to the implant. Because of the lateral extra-articular approach used for implants, only a distal minimal incision (described above) is needed, which has the clinical benefit of foregoing much higher disruption and morbidity associated with transosseous fixation.

**Figure 5:**
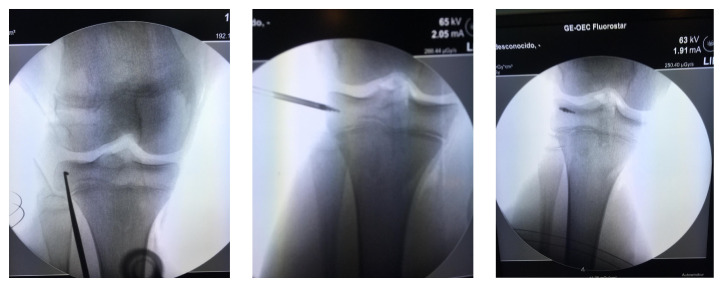
ACL reconstruction on physis open patients. Under fluoroscopic control on the operating, selection of the footprint and intruction the TWINFIX for LET reconstruction.

This technique also has the advantage that it can be used in pediatric patients, with the anchor implant placement achieved by radiographic control with a minimal incision and placing the anchor in the correct epiphyseal location to avoid injury of the cartilage growth zone and preserve the integrity of the bone growth plate (Figure 5). All patients returned to their usual work and sports activities without incidence of device-related or serious adverse events. In all cases, recovery of joint stability was achieved and complete recovery of full range of motion, except for the patient who underwent the manipulation under anesthesia who had a persistent 10° degree extension deficiency.

In the future, the possible development of non-metallic implants may help to avoid magnetic resonance imaging interference and more closely match the mechanical and biological properties of bone. The current anchor product allows for achieving accurate and safe repair, with fixation of the knee tendons on bone (in this situation combining different repair techniques) in a similar fashion to other joints such as the shoulder [18].This is possible because the anchor design provides the necessary stiffness and stability at the anchor implantation site in bone, allows early local biological response, and bone in-growth.

As previously described, the LET supports greater improvement for the rotational stability of the knee in the context of ACL reconstruction, and should result in a greater survival of the graft (especially in patients with a high functional demand, hyperlaxity or young patients) [14,21]. These techniques require drilling of bone tunnels on the tibial metaphysis or the use of more aggressive implants, [22] such as interference screws [23] or implants with greater morbidity (e.g., staples), which were used by the author before and in many cases required extraction due to the inconvenience they cause. Therefore, we consider this type of suture to offer advantages in allowing minimally invasive surgery at the incision site, with mechanical properties than confer an agreeable safety profile.

In our practice, we prefer the use of this type of implant to using a linear strip of the iliotibial band, which could weaken the lateral area of the knee, especially if the Kaplan fibers are damaged.

The limitations of this technique were the possibility of presence of more implications in the evaluation’s methods, as for the ligament injury, the affected range of mobility because of the affected tendons or the presence of osteoarthritis previously to the surgery.

## 5. Conclusions

In conclusion, this retrospective case series supports the safety and performance of these implants for the knee procedures in which its use is indicated used the KOOS outcome measure. Additional follow-up will be required to determine whether these effects are sustained at medium- and long-term durations.

## Figures and Tables

**Table 1:** Patients’ preoperative and postoperative KOOS score.

	Mean KOOS Score (SD)		*p*-Value	ES Cohen’s d
	Preoperative	Postoperative		
Pain	52 (19.2)	87.3 (10.3)	<0.001	0.6
Symptoms	53.9 (16.3)	78.4 (9.9)	<0.001	0.5
Funtion of daily living	48.3 (20.2)	90.4 (8.6)	<0.001	0.7
Recreation Function	38.4 (23.2)	75.6 (10.1)	<0.001	0.7
Quality of life(QoL)	32.7 (23.7)	57.9 (18.3)	<0.001	0.5

SD standard deviation, ES effect size.

## Data Availability

The data that support the findings of this study are available from the corresponding author upon reasonable request.
